# AtHB7/12 Regulate Root Growth in Response to Aluminum Stress

**DOI:** 10.3390/ijms21114080

**Published:** 2020-06-07

**Authors:** Yang Liu, Jiameng Xu, Siyi Guo, Xianzheng Yuan, Shan Zhao, Huiyu Tian, Shaojun Dai, Xiangpei Kong, Zhaojun Ding

**Affiliations:** 1 The Key Laboratory of Plant Development and Environmental Adaptation Biology, Ministry of Education, College of Life Sciences, Shandong University, Qingdao 266237, China; 201920296@mail.sdu.edu.cn (Y.L.); jmengxu@126.com (J.X.); tianhuiyu@sdu.edu.cn (H.T.); 2 The Key Laboratory of Plant Stress Biology, School of Life Science, Henan University, JinMing Avenue, Henan University, Kaifeng 475004, China; guosiyi@henu.edu.cn; 3 Shandong Key Laboratory of Water Pollution Control and Resource Reuse, School of Environmental Science and Engineering, Shandong University, Qingdao 266237, China; xzyuan@sdu.edu.cn (X.Y.); szhao@sdu.edu.cn (S.Z.); 4 Development Center of Plant Germplasm Resources, College of Life Sciences, Shanghai Normal University, Shanghai 200234, China; daishaojun@hotmail.com

**Keywords:** root, aluminum stress, AtHB7/12, yeast two hybrid, HD-Zip I transcription factors

## Abstract

Aluminum (Al) stress is a major limiting factor for plant growth and crop production in acid soils. At present, only a few transcription factors involved in the regulation of Al resistance have been characterized. Here, we used reversed genetic approach through phenotype analysis of overexpressors and mutants to demonstrate that AtHB7 and AtHB12, two HD-Zip I transcription factors, participate in Al resistance. In response to Al stress, *AtHB7* and *AtHB12* displayed different dynamic expression patterns. Although both AtHB7 and AtHB12 positively regulate root growth in the absence of Al stress, our results showed that AtHB7 antagonizes with AtHB12 to control root growth in response to Al stress. The *athb7/12* double mutant displayed a wild-type phenotype under Al stress. Consistently, our physiological analysis showed that AtHB7 and AtHB12 oppositely regulate the capacity of cell wall to bind Al. Yeast two hybrid assays showed that AtHB7 and AtHB12 could form homo-dimers and hetero-dimers in vitro, suggesting the interaction between AtHB7 and AtHB12 in the regulation of root growth. The conclusion was that AtHB7 and AtHB12 oppositely regulate Al resistance by affecting Al accumulation in root cell wall.

## 1. Introduction

A major factor constraint crop production on acidic soils worldwide is aluminum (Al) toxicity [1]. Excess Al^3+^ in acid soils is toxic to plants. It has been reported that the rapid inhibition of root elongation is the earliest and most dramatic symptom of Al toxicity, resulting in a reduced and damaged root system that limits mineral nutrient and water uptake, and eventually leads to reduced production [2,3,4,5].

The root transition zone (TZ), located between the apical meristem and basal elongation zone (EZ) in root apex, is the critical site of perception of Al toxicity, which has been reported in the model plant Arabidopsis (*Arabidopsis thaliana*), wheat (*Triticum aestivum*), maize (*Zea mays*), sorghum (*Sorghum bicolor*), and common bean (*Phaseolus vulgaris*) [6,7,8,9,10,11]. The root TZ has also been thought to be the active site for phytohormone cross-talks. Al stress induced local auxin biosynthesis through the up-regulation of *TAA1* and *YUCCAs.* The local accumulated auxin in root TZ further increased local cytokinin biosynthesis and enhanced cytokinin responses and eventually inhibited root growth, a process which was dependent on ethylene signaling [7,12,13]. However, in maize, external supply of auxin to the root EZ is able to partly overcome the inhibition of root under Al stress, indicating the repressed root growth might be result from low auxin accumulation in root tips [14]. This hypothesis was further confirmed by characterization of the *zmpgp1* mutants which are deficient in the maize auxin efflux carrier P-glycoprotein (ZmPGP1) and displayed higher levels of auxin in root tips and increased Al tolerance [15]. All these investigations suggest that auxin plays an important role in root growth regulation under Al stress.

When plants suffer Al toxicity, cell wall is the main site for Al accumulation. For example, 80%–90% of Al accumulated in the cell wall of barley [16]; in total, 95% of Al deposited in the cell wall of hypocotyl epidermal cells in *Abelmoschus esculentus* [17]. It has been widely accepted that the large amount of Al deposited in the cell wall alters its property, reduces its ductility and arrests cell expansion in root elongation zone [18], which are the main causes of Al-induced inhibition of root growth. Pectin methylation and modifications of xyloglucan oligosaccharides which are the major components of cell wall hemicellulose in non-poalean monocotyledons and dicotyledons, are recognized as two vital strategies to affect cell wall Al binding capacity. PME46 (PECTIN METHYLESTERASE46), an inhibitor of pectin methylesterase activity, increased methylated pectin and reduced Al binding to cell wall and thus conferred plants Al resistance [19]. Besides, Al binding capacity of hemicelluloses could be positively regulated by xyloglucan endotransglucosylase/hydrolases AtXTH17 and AtXTH31, and accordingly, lacking each of the XTH enzymes leads to increased Al-sensitivity [20,21]. Mutants of *TRICHOME BIREFRINGENCE-LIKE27* (*BL27*), encoding a putative *O*-acetyltransferase for modulation of the O-acetylation level of xyloglucan, showed increased Al sensitivity by enhancing Al-binding in the hemicellulose [21]. In rice (*Oryza sativa*), *Arabidopsis* (*Arabidopsis thaliana*) and buckwheat (*Fagopyrum esculentum*), STAR1 interact with STAR2/ALS3 to form an ABC transporter to regulate Al resistance possibly via transporting UDP-glucose for cell wall modification [22,23,24,25,26]. Knockout of *OsEXPA10* in rice affected Al binding to cell wall, but did not affect Al-induced root-growth inhibition [27].

Besides modifying cell wall, plants have also employed other strategies to encounter with Al toxicity, such as organic acid exudation and internal detoxification like vacuolar compartment [28]. Normally, few malic acid and citric acid were secreted from the roots. However, in response to Al stress, both *AtALMT1* (*Al-activated Malate Transporter1*) and *AtMATE* (*Multidrug and Toxic Compound Extrusion*) were significantly up-regulated at transcription levels, which caused the secretion of malic acid and cirtic acid into rhizosphere for Al chelation, and thus alleviated Al-induced inhibition of root elongation [29,30]. Furthermore, ALS1 (ALUMINUM SENSITIVE1), which located in tonoplast, seemed to participate in transporting Al to vacuole for segregation [31].

In *Arabidopsis thaliana*, SENSITIVE TO PROTON RHIZOTOXICITY1 (STOP1), a type of C2H2 zinc finger transcription factor, was identified as a key regulator in response to Al stress through the transcriptional regulation of *AtALMT1*, *AtMATE*, and *ALS3*, which encoded organic acids transporters and a half-type ABC transporter, respectively [32,33]. Subsequently, orthologous genes have been identified in rice and sweet sorghum [34,35,36]. *STOP2* is a homolog of *STOP1*, having a shorter C-terminus compared to STOP1. AtSTOP2 activates the expression of some genes involved in Al- and low pH-tolerance that are also regulated by AtSTOP1 such as *AtMATE* and *AtALS3* [37]. Recently, an F-box protein encoded by the gene regulating *AtALMT1* expression 1 (RAE1) was shown to regulate STOP1 protein abundance. The RAE1 protein interacts with STOP1 and hence promotes STOP1 ubiquitination and degradation [38]. AtWRKY46 was reported as a negative regulator of *AtALMT1* transcription to restrain malate secretion under Al stress [39]. AtWRKY47 directly regulated *ELP* (*EXTENSIN-LIKE PROTEIN*) and *XTH17* which are responsible for cell wall modification under Al stress [40]. As a STOP1 ortholog in rice, ART1 functions as a central modulator of Al response, regulating expression of a broad of crucial Al resistance genes, including *OsSTAR1/2*, *OsNrat1*, *OsALS1*, *OsFRDL4*, *OsMGT1* and *OsCDT3* [34]. ART2, a homology of ART1, plays a supplementary role in Al tolerance in rice. [35]. OsWRKY22 was involved in regulation of Al-activated citrate acid secretion by regulating expression of *OsFRDL4* which encodes a citrate transporter in rice [41]. ABSCISIC ACID, STRESS AND RIPENING (ASR) transcription factor ASR5 collaboratively acts with ASR1 in Al-stress response [42,43].

HD-ZIP transcription factors which are unique in plants are characterized as containing a homeodomain (HD) for binding DNA and a leucine zipper (Zip) motif for protein dimerization [44]. HD-ZIP I proteins from different species were reported to participate in various abiotic adversity responses. In rice, OsHOX22 acted as a negative regulator of drought and salt tolerance through an ABA-dependent pathway [45]. MtHB1 from *Medicago truncatula* controlled lateral root emergence under abiotic stresses [46]. ZmHDZ10 positively regulated drought and salt stress dependent on ABA signal pathway [47]. In *Arabidopsis*, AtHB6 was reported to be involved in ABA response by interacting with the PP2C phosphatase ABI1 and mediated drought stress responses acting downstream of ABI1 and ABI2 [48,49]. AtHB13 regulated response to freezing temperature via inducing genes expression which involved in cell membrane stabilization and inhibition of ice growth [50]. In addition, AtHB13 positively regulates a NAC transcription factor gene *JUB1* expression to confer *Arabidopsis* drought tolerance [51]. AtHB7 and AtHB12 negatively module ABA signaling pathway by activating *PP2C* genes and repressing ABA receptors *PYL5* and *PYL8* [52]. Olsson et al. showed that AtHB7 and AtHB12 are negative developmental regulators in response to drought [53]. Our previous data showed that *AtHB7* and *AtHB12* were highly up-regulated among the differential expression genes in Col-0 wild type treated with or without Al [7].

Since AtHB7 and AtHB12 as two members of HD-ZIP I subfamily play important roles in regulation of tolerance to abiotic stress and were significantly induced by Al, we speculated that these two transcription factors might relate to regulation of Al resistance. Here, we showed that AtHB7 and AtHB12 acted in a cooperative manner under normal condition but in an antagonistic manner to regulate primary root growth under Al treatment.

## 2. Results

### 2.1. AtHB7 and AtHB12 Induced by Al Stress, Which Was Independent of STOP1

**Figure 1:**
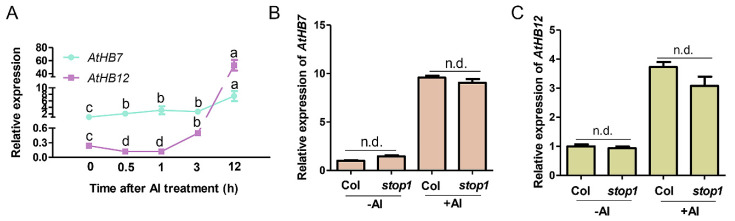
Two HD-Zip I transcription factors *AtHB7* and *AtHB12* response to Al stress independent of STOP1 regulation. (**A**) Time-course analysis of *AtHB7* and *AtHB12* relative expression in roots in response to 20 μM Al exposure. The relative expression was normalized to *ACTIN2* expression and *AtHB7* expression untreated with Al was set as 1. Relative expression of *AtHB7* (**B**) and *AtHB12* (**C**) in roots of wild type and *stop1* mutant in response to 20 μM Al exposure. Data are means ± SD (*n* = 3). Different letters indicate significant differences by Tukey’s test.

*AtHB7* and *AtHB12*, the most closed members in HD-ZIP I subfamily, were up-regulated 13 folds and 206 folds respectively by Al treatment [7]. To characterize the potential roles of *AtHB7* and *AtHB12* in root growth response to Al stress, we further analyzed the expression patterns of *AtHB7* and *AtHB12* under Al stress through a time-course analysis. Real-time qPCR results showed that *AtHB7* mRNA level was dominant in early response stage and increased with Al treatment, whereas *AtHB12* expression level appeared to be down-regulated initially, and then up-regulated at 6 h after Al exposure and surpassed *AtHB7* accumulation level at 12 h (Figure 1A). The different dynamic expression of *AtHB7* and *AtHB12* in response to Al stimuli suggests that AtHB7 and AtHB12 might play different roles in response to Al stress. To clarify the molecular mechanism of transcriptional regulation of *AtHB7* and *AtHB12* in response to Al stress, we firstly examined if STOP1, which is a key transcription factor during Al stress response and regulates multiple Al resistance genes expression, was involved in this process. The results showed that the expression patterns of *AtHB7* and *AtHB12* were not changed in *stop1* mutant with or without Al treatment, indicating that Al-regulated *AtHB7* and *AtHB12* expression is independent of STOP1 (Figure 1B,C).

**Figure 2:**
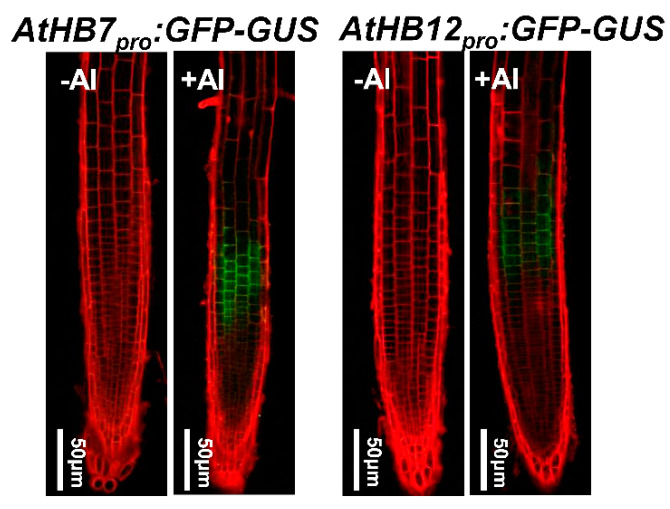
Both *AtHB7* and *AtHB12* were induced by Al toxicity in transition zone. Expression of *AtHB7pro:GFP-GUS* and *AtHB12pro:GFP-GUS* in root tips of seedlings exposed without or with 20 μM Al for 6 h were analyzed by observation using confocal laser scanning microscopy.

To address the expression patterns of *AtHB7* and *AtHB12* in the presence of Al stress, the effect of exposure to Al stress on the spatial expression of *AtHB7* and *AtHB12* was analyzed by monitoring the expression of the *AtHB7pro:eGFP-GUS* and *AtHB12pro:eGFP-GUS* transgenes. After a 6 h exposure to 25 µM Al, GFP signals were highly detected in the root apex TZ, while there is no GFP signal in the root tips under non-stressed conditions (Figure 2).

### 2.2. AtHB7 and AtHB12 Promote Root Growth by Regulating Cell Number and Cell Length

**Figure 3:**
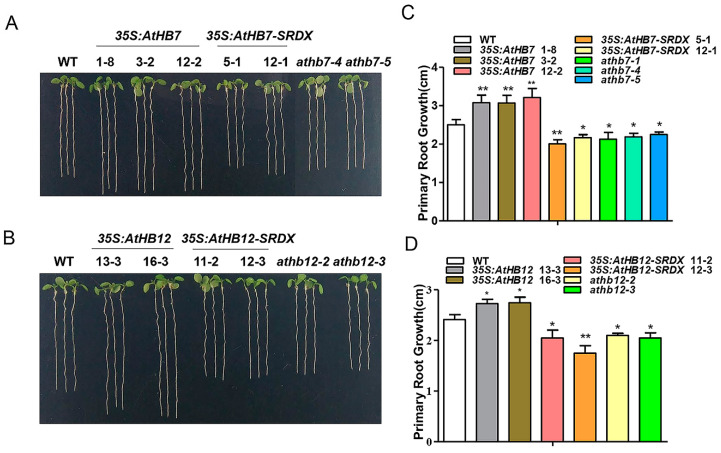
AtHB7 and AtHB12 contribute to primary root growth during normal development. Phenotype of 6-day-old *AtHB7* (**A**) and *AtHB12* (**B**) overexpression lines, SRDX (SUPERMAN repression domain) transgenic seedlings and mutants under standard growth condition. Primary root length in A (**C**) and B (**D**). Data are means ± SD (*n* = 24). Asterisks indicate significant differences between wild type and transgenic lines at *: *p* < 0.05 and **: *p* < 0.01 by Tukey’s test.

**Figure 4:**
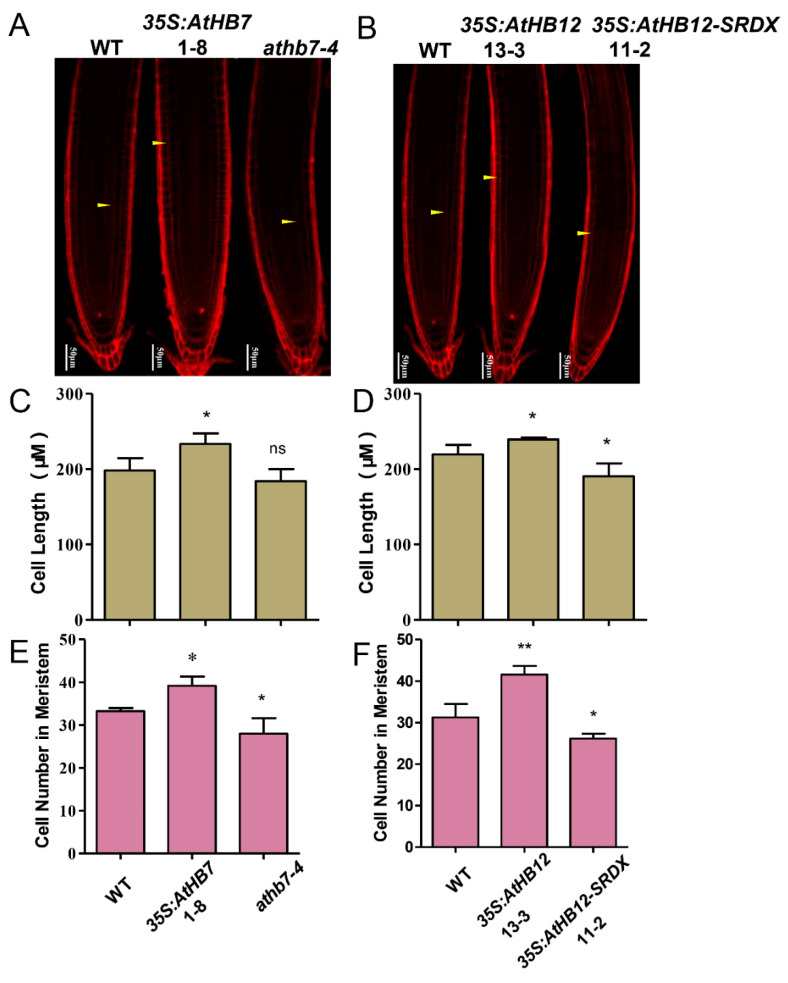
AtHB7 and AtHB12 module root growth by regulating cell number and cell length. Confocal images of root tips of 6-day-old wild type, *AtHB7* (**A**) and *AtHB12* (**B**) transgenic seedlings including overexpression, SRDX and CRISPR (Clustered regularly interspaced short palindromic repeats) lines stained with PI. White Arrows indicated the first elongated cell in cortex. Cell length of elongation zone in *AtHB7* (**C**) and *AtHB12* (**D**) transgenic plants. Cell number in RAM (root apical meristem) of *AtHB7* (**E**) and *AtHB12* (**F**) transgenic plants. Data are means ± SD (*n* = 30). Asterisks indicate significant differences between wild type and transgenic seedlings at *: *p* < 0.05 and **: *p* < 0.01 by Tukey’s test.

To investigate whether Al responsive *AtHB7* and *AtHB12* are involved in root growth, we generated *35S:AtHB7/12* overexpression transgenic plants, the dominant-negative constructs *35S:AtHB7/12-SRDX* (SUPERMAN repression domain, SRDX) lines and *athb7* and *athb12* mutants edited by CRISPR CAS9 system (Supplementary Figure S1). Under normal condition, the roots of *35S:AtHB7* and *35S:AtHB12* were longer than those of the wild type, while *35S:AtHB7-SRDX* and *35S:AtHB12-SRDX* showed short-root phenotypes, which were also confirmed by root length analysis in the *athb7* and *athb12* mutants (Figure 3). A longitudinal zonation pattern analysis showed that root apical meristem (RAM) of *35S:AtHB7* and *35S:AtHB12* seedlings were significantly longer than that of the WT control (Figure 4A,B). Cell numbers in the meristem zone (MZ) and cell length in the elongation zone (EZ) of *35S:AtHB7* and *35S:AtHB12* seedlings were both increased compared to the WT control (Figure 4C–F). However, the *athb7* mutant and the *35S:AtHB12-SRDX* transgene showed a reduced size in the root MZ, and cortical cells in the EZ were shorter than those in WT, and cell numbers in the MZ were also strongly reduced (Figure 4C–F). These results suggest that AtHB7 and AtHB12 affect both cell proliferation and cell elongation.

### 2.3. AtHB7 and AtHB12 Antagonistically Regulate Root Elongation under Al Stress

**Figure 5:**
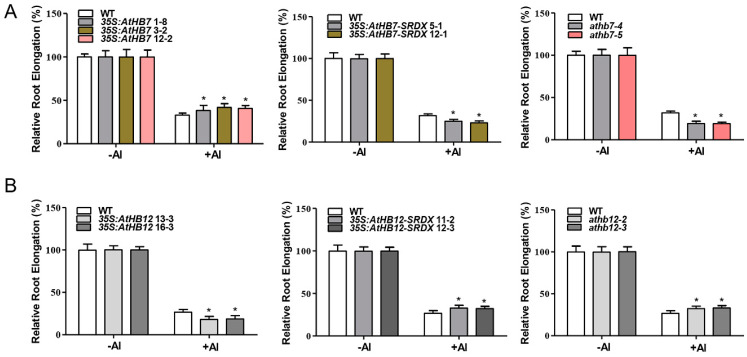
AtHB7 positively regulates Al resistance (**A**), whereas AtHB12 negatively regulates Al resistance (**B**). Relative root elongation of wild type, *AtHB7* (**A**) and *AtHB12* (**B**) overexpression, SRDX and CRISPR lines in response to 6 μM Al for 6 days. Data are means ± SD (*n* = 30). Asterisks indicate significant differences between wild type and transgenic seedlings at *: *p* < 0.05 by Tukey’s test.

We further examined the root growth under Al stress. The results showed that *35S:AtHB7* transgenic lines displayed a reduced root-growth inhibition in response to Al stress, while *35S:AtHB7-SRDX* transgenic lines and *athb7* mutants all showed an enhanced root-growth inhibition after Al treatment (Figure 5A). However, there were no difference between wild type and the *35S:AtHB7*, *35S:AtHB7-SRDX* transgenic lines and *athb7* mutants when plants were exposed to solutions containing different toxic ions including sodium (Na^+^), copper (Cu^2+^), lanthanum (La^3+^) and cadmium (Cd^2+^), or to various pH solutions (4.5–6.5), indicating that AtHB7 responds to Al stress specifically (Supplementary Figure S2). These results indicate that AtHB7 is a positive regulator of Al resistance. In contrast to *AtHB7*, *35S:AtHB12* transgenic lines showed an elevated root growth inhibition in response to Al stress, while *35S:AtHB12-SRDX* and *athb12* mutants showed significantly less inhibition than the WT control (Figure 5B). Similar to AtHB7, no differences in root growth were observed when WT and *35S:AtHB12*, *35S:AtHB12-SRDX* transgenic lines and *athb12* mutant plants were exposed to other metal ions stress and different pH solutions (Supplementary Figure S3). These results indicate that AtHB12 negatively regulates Al resistance.

### 2.4. AtHB7 and AtHB12 Oppositely Regulate Aluminum Deposition in Cell Wall

**Figure 6:**
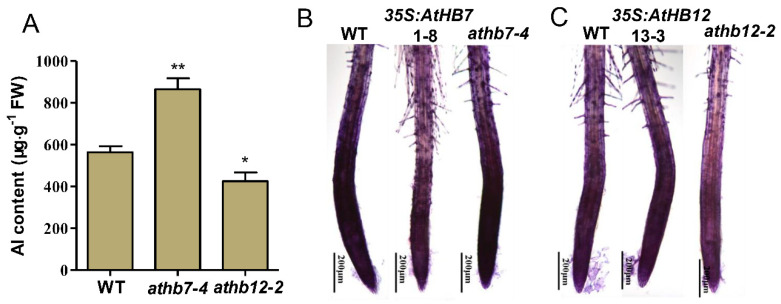
AtHB7 and AtHB12 oppositely regulate Al binding to cell wall. (**A**) Analysis of Al content in roots of wild type, *athb7* and *athb12* mutants. AtHB7 (**B**) and AtHB12 (**C**) affect Al accumulations in cell walls of root tips indicated by hematoxylin staining. Asterisks indicate significant differences between wild type and mutants at *: *p* < 0.05 and **: *p* < 0.01 by Tukey’s test.

To investigate the underlying mechanisms of how AtHB7/12 regulate Al resistance, Al content in roots of *athb7* mutant, *athb12* mutant and wild type was detected. Compared with wild type, *athb7* mutant accumulated much more Al in roots, but *athb12* mutant held less Al in roots (Figure 6A). In depth, hematoxylin staining technique was used to visualize Al accumulation in root cell walls of wild type, *AtHB7* or *AtHB12* transgenic plants. The staining intensity was stronger in *athb7* root tip region and decreased in *AtHB7* overexpression plants compared to wild type (Figure 6B). However, stronger intensity in *AtHB12* overexpression plants and weaker stain in the *athb12* mutant were observed (Figure 6C). Taken together, these results indicate that AtHB7 and AtHB12 differently regulated Al tolerance by affecting Al accumulation in root cell wall.

### 2.5. AtHB7 and AtHB12 Might Act in Homodimer or Heterodimer to Control Root Growth under Al Stress

**Figure 7:**
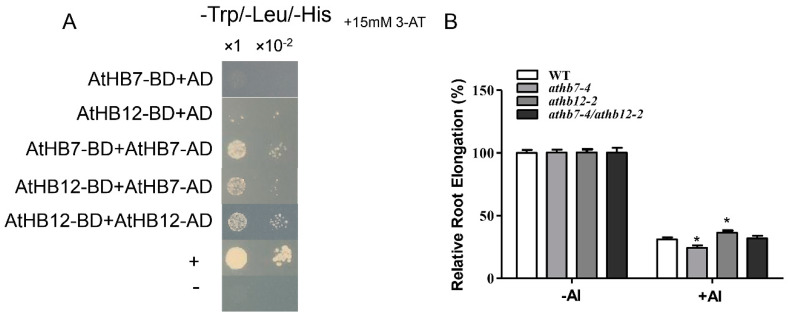
AtHB7 and AtHB12 might act in homodimer or heterodimer in response to Al stress. (**A**) AtHB7 and AtHB12 could form homodimer and heterodimer showed by yeast two-hybrid assay. (**B**) Relative root growth of wild type, *athb7*, *athb12* and *athb7/athb12* double mutant in response to Al treatment. Data are means ± SD (*n* = 30). Asterisks indicate significant differences between wild type and mutants at *: *p* < 0.05 by Tukey’s test.

HD ZIP transcription factors are known for containing a homeodomain/leucine zipper domain, the former domain specifically binding to DNA and the latter domain mediating the formation of protein dimers. Based on the high level homology between AtHB7 and AtHB12 and the distinctively opposite function in Al-stress response, we speculated that AtHB7 and AtHB12 may interact to form a heterodimer that affects protein activity or stability to antagonistically regulate Al resistance in *Arabidopsis*. Yeast two-hybrid assays showed that AtHB7 could not only form homodimer with itself but also form heterodimer with AtHB12 (Figure 7A). Further, the *athb7/hb12* double mutant exhibited similar Al resistance with wild type (Figure 7B). These results suggest that AtHB7 and AtHB12 may form a heterodimer to inhibit mutual activities in regulating downstream Al-response genes under Al stress.

## 3. Discussion

Al stress in one of the most limiting factors for plant growth and crop productivity in acidic soils. Identification of the molecules involved in Al tolerance mechanisms is a current challenge for Al toxicity. Previous studies identified STOP1 as a key transcription factor that regulates primary root growth in response to Al stress. In the present study, we identified two novel regulators of Al resistance. AtHB7 and AtHB12, two HD-Zip I transcription factors, oppositely regulate Al resistance in *Arabidopsis.* This is directly proved by the fact that *AtHB7* overexpression lines and *athb12* mutants are tolerant to Al and *athb7* mutant, and *AtHB12* overexpression lines are sensitive to Al (Figure 5). However, *AtHB7* and *AtHB12* are not associated with tolerance to other metal such as La^3+^, Cd^2+^, Cu^2+^, Na^+^ and low pH (Supplementary Figures S2 and S3). Therefore, we conclude that AtHB7 and AtHB12 are specifically involved in Al resistance. The HD-ZIP transcription factor family consists of 47 members in *Arabidopsis* and several HD-ZIP I subfamily members were found to take part in response to unfavorable conditions such as salt stress, drought stress, ABA and cold stress. In the study, we firstly demonstrated that these two proteins belonging to HD-ZIP I subfamily function in Al resistance mechanisms.

Although AtHB7 and AtHB12 are the most closely related members in HD-Zip I family (Supplementary Figure S4), AtHB7 and AtHB12 showed opposite function in Al resistance. Previous study also showed that *AtHB7* was repressed by water loss, while *AtHB12* was induced by water loss under water stress conditions; in addition, AtHB7 and AtHB12 play different roles in certain biological process or developmental stage like stomata aperture and senescence [54].

It is interesting to note that AtHB7 and AtHB12 play opposite roles in Al resistance, while both promote root growth during normal development (Figure 3 and Figure 5). The difference of DNA-binding homeodomains between AtHB7 and AtHB12 might lead to distinct target genes in response to Al stress; AtHB7 and AtHB12 also perhaps interact with different partners in Al-response due to the distinct leucine zipper domains between AtHB7 and AtHB12. For instance, some transcription factors interact with transcriptional repressor to negatively regulate target genes. Human transcription factor LEF1 was showed to interact with HDAC1 (Histone Deacetylase1) and its function shift from activation to repression regulated by β-Catenin–Histone Deacetylase [55]. BZR1 acts as either a transcriptional activator or a repressor partly depend on the context of promoters of target genes and *trans*-factors. BZR1-PIF4 (phytochrome interacting factor 4) heterodimer binds to the E-box motif to activate target expression [56]; while BZR1 interacts with TPL (Groucho/TUP1-like corepressor TOPLESS) through its EAR motif to transcriptionally repress target genes [57]. AtHB7 and AtHB12 were revealed to function as transcription activators in *Arabidopsis* under normal conditions by transient expression assays [44]. However, recent report showed that AtHB12 interacted with TFIIB through its AHA (aromatic large hydrophobic acidic residues) transactivation motif which locates in carboxy termini, while AtHB7 interacted with both TBP and TFIIB to activate target genes [58].

Our results showed that AtHB7 and AtHB12 were able to form heterodimers (Figure 7A). The leucine zipper domain of HD Zip provides proper structure to allow the formation of dimers [59,60]. For example, AtHB5 was reported to be able to form homodimer and heterodimers with other HD-Zip I members [61]. OsHox1 as a member of HD-Zip Ⅱ family could interact with HAT which is an *Arabidopsis* HD-Zip I member, whereas the corresponding overexpression transgenic plants show opposite phenotype such as developmental rate [62]. Hetero-dimerization of AtHB7 and AtHB12 seems to inhibit activities of each other under Al stress, and the *athb7/hb12* double mutant show similar Al resistance with wild type (Figure 7B).

In this study, we further demonstrated that AtHB7 and AtHB12 differently mediated Al-induced root elongation, which was attributed to distinct capacity of cell wall to bind Al (Figure 6). It is in line with previous findings that AtHB12 regulates expression of cell wall associated genes. *AtHB12* overexpression promotes leaf cell expansion by increasing expression of cell wall-related genes such as *EXPANSINA10* and *DWARF4* [63]. Cell wall is composed of pectin, hemicellulose and cellulose, and pectin and hemicellulose were accepted as the major targets to bind Al [64,65,66]. It is well known that compositional and structural modification of the cell wall, especially pectin methylation and modification of xyloglucan oligosaccharides, exert crucial function in Al resistance. Functional genes associated with cell wall modification were widely characterized in *Arabidopsis* such as *WAK1* (*cell wall-associated receptor kinase 1*), *PME46*, *LUH* (*LEUNIG_HOMOLOG*), *STAR1* (*Sensitive to Al rhizotoxicity1*), *ALS3* (*ALUMINUM SENSITIVE 3*), and *XTH17* and *XTH31* [19,20,22,23,24,25,67,68]. Next, we intend to explore whether the above genes mediated AtHB7/12 regulated Al resistance, and discover more novel Al-resistance genes controlled by AtHB7 or AtHB12 based on whole transcriptome sequencing to enrich our knowledge in mechanisms of plants Al resistance.

## 4. Materials and Methods

### 4.1. Plant Culture and Treatment

Arabidopsis cultured in Petri dishes with 1/2 MS solid medium or in nutrient solutions or in soils were placed in a plant growth chamber at 22 °C, 150 μmolm^−2^ s^−1^ light intensity with a long-day photoperiod (16-h light/8-h dark). For plate culture, seeds were surface disinfected with 10% sodium hypochlorite solution for 5 min, and then washed three times with sterile water [69]. The disinfected seeds were sowed onto 1/2 MS medium and incubated at 4 °C in darkness for 2 days. Then, the plates with seeds were vertically placed in the chamber. For Al and different metal treatment, Arabidopsis seeds soaked in sterile water at 4 °C overnight germinated in 2% MGRL solution (pH 5.0) without or with 6 μM AlCl_3_, 1 mM LaCl_3_, 3.5 mM CdCl_2_, 2 mM CuCl_2_, 10 mM NaCl for 6 days. For different pH treatment, the soaked Arabidopsis germinated in 2% MGRL solution with pH 6.5, 6.0, 5.5, 5.0, 4.7, 4.5 for 6 days. Treatment solution was renewed every day. Then, the primary root length was analysis by Image J (developed by National Institutes of Health) after treatment.

### 4.2. Materials and Transgenic Arabidopsis Construction

*athb7* and *athb12* mutants were obtained by YAO-promoter driven CRISPR/cas9 gene editing method according to previous reports [70]. Briefly, two sgRNA targets of each gene were designed, then cloned into middle vector in turn and eventually two *pYAO:hSpCas9-target1-sgRNA-target2-sgRNA* recombinant vectors for editing *AtHB7* and *AtHB12* respectively were constructed. The amplified *AtHB7* and *AtHB12* ORF were respectively integrated into pENTR^™^ TOPO^®^ vector using pENTR™/D-TOPO™ Cloning Kit (Thermo Fisher, catalog number K2400-20), and subsequently transferred to pB7WG2 Gateway destination vector to obtain *35S:AtHB7* and *35S:AtHB12* overexpression vectors. *AtHB7* and *AtHB12* coding sequences were respectively fused with the EAR-repression domain (SRDX) under the control of the *35S* promoter to generate *35S:AtHB7-SRDX* and *35S:AtHB12-SRDX* recombinant vectors. In total, 2.1 Kb DNA fragment upstream from initiation codon of *AtHB7* was cloned to drive repoter genes expression using Gateway cloning technology to construct *AtHB7pro:eGFP-GUS*. Likewise, approximate 2.2 Kb promoter sequence of *AtHB12* was amplified to obtain *AtHB12pro:eGFP-GUS* plasmid.

The above final expression vectors were separately transformed into Col-0 wild type by floral dip method. Then, three T3 homozygous overexpression lines *35S:AtHB7#1*(1-8), *#2*(3-2), *#3*(12-2), two AtHB7-SRDX transgenic lines *35S:AtHB7-SRDX#1*(5-1), *#2* (12-1) and three CRISPR/Cas9-induced *athb7* mutant plants *athb7-1*, *-4*, *-5* were used for research. To explore AtHB12 gene function, two homozygous overexpression lines *35S:AtHB12#1*(13-3), *#2*(16-3), two SRDX lines *35S:AtHB12-SRDX#1*(11-2), *#2* (12-3) and two *athb12* mutants *athb12-2*, *-3* were employed in this study. *athb7-4/athb12-2* double mutant was generated by cross *athb7-4* (male parent) with *athb12-2* (female parent). The homozygous offerings were identified with PCR. All primers used for recombinant constructions and identification of different genotype were listed in Supplementary Table S1. 

### 4.3. Phylogenetic Analysis

The amino acid sequences of HD-ZIP I members were retrieved from TAIR database and then were aligned with Clustal 2.0 software [71]. The phylogenetic tree was constructed using MEGA 7.0 by neighbor-joining (NJ) method with 1000 bootstrap replications [72].

### 4.4. RNA Isolation and qRT PCR

Total RNA was extracted from intact roots of 6-day-old plants treated without or with 20 μM Al using TaKaRa MiniBEST Plant RNA Extraction Kit (TAKARA, catalog number 9769). At first, genome DNA was removed by gDNase (TIANGEN BIOTECH Co., Ltd., catalog number KR116) (TIANGEN Biotech, Beijing, China). Then, 1 μg RNA was synthesized into the first cDNA strand with FastKing RT Kit according to manufacturers’ protocols (TIANGEN BIOTECH Co., Ltd., catalog number KR116) (TIANGEN Biotech, Beijing, China). The real-time quantitative RT-PCR was performed with SuperReal PreMix Plus (SYBR Green) provided by TIANGEN company using CFX Connect Real-Time System (Bio-Rad, Hercules, CA, USA) [73]. qPCR reaction conditions were 45 cycles at 95 °C for 10 s, 56 °C for 20 s, and 72 °C for 20 s. Expression levels were normalized to the expression level of *UBQ10*. All qRT PCR experiments were done three repeats from different biological samples.

### 4.5. Confocal Microscopy

6-day-old AtHB7pro:eGFP-GUS or AtHB12pro:eGFP-GUS seedlings were treated in 2% MGRL solution without or with 20 μM Al at pH 5.0. Then, the roots were immersed in 1 μg/ml propidium iodide (PI) solution and imaged using a confocal laser scanning microscope (Zeiss LSM700). For PI and GFP channels, the 543 nm and the 488 nm wavelengths were used for excitation, respectively. Four-day-old seedlings of wild type, AtHB7 or AtHB12 transgenic plants were stain with PI for growth analysis. Cortical cell numbers in the root apical meristem (RAM) and length of elongation zone cell were determined as previous described [74]. In detail, RAM comprised of the organized cells from QC to the first elongated cell. Elongation zone boundaries were from the first dramatically elongating cell to the cell developing root hairs.

### 4.6. Al Content Determination

Seeds of wild type, *athb7* and *athb12* mutants germinated in 2% MGRL solution at pH 5.5 for 6 days and then the seedlings were exposed to 2% MGRL solution with 10 μM Al at pH 5.0. After 24 h, the roots were excised, washed three times with deionized water, dried and digested with 65% HNO_3_. Al content in roots was measured by GF-AAS (SHIMADZU, Japan).

### 4.7. Hematoxylin Staining

Arabidopsis seeds were germinated in 2% MGRL solution at pH 5.5 for 6 days and then the seedlings were transferred to 2% MGRL solution supplemented with 20 μM Al at pH 5.0 for 6 h. After treatment, the seedlings were stained with 0.2% hematoxylin containing 0.02% KIO_4_ for 20 min and washed three times with deionized water before observations. The samples were observed and photographed using an Olympus BX53 microscope equipped with an Olympus DP72 camera system.

### 4.8. Yeast Two-Hybrid Assay

Analysis of protein–protein interactions in yeast were carried out using the Matchmaker Gold Yeast Two-Hybrid System provided by TAKARA. The coding sequence of AtHB7 and AtHB12 were transferred from pENTR^TM^ to the pGBKT7 (BD) vector by Gateway LR reaction to generate the constructs AtHB7-BD and AtHB12-BD as the preys. Similarly, the amplified coding sequence of AtHB7 and AtHB12 were fused with the PGADT7 (AD), resulting in the baits. The prey and bait were co-transformed into yeast cells. The transformed yeasts were selected on dropout medium without Leu and Trp. Then analysis of interaction was indicated by the growth of yeast colonies on synthetic medium lacking Leu, Trp, His or lacking Leu, Trp, His, and Ade with 3-AT incubated at 30 °C for 3 days.

### 4.9. Statistical Analysis

Statistical analysis was performed by Tukey’s test among treatments. Three biological replicates for each treatment were conducted for the statistical analysis in this article. Asterisks or different letters in the figures indicated significant differences as follows: * *p* < 0.05, ** *p* < 0.01, and *** *p* < 0.001.

## Supplementary Materials

Supplementary materials can be found at https://www.mdpi.com/1422-0067/21/11/4080/s1, Figure S1: Structure of mutated *AtHB7* and *AtHB12* genes edited by Crispr/Cas9. Figure S2: AtHB7 specifically regulates Al resistance. Relative root elongation of wild type, *AtHB7* transgenic seedlings including overexpression, SRDX and CRISPR lines in response to different metal ions and pH changes. Figure S3: AtHB12 specifically regulates Al resistance. Relative root elongation of wild type, *AtHB12* transgenic plants including overexpression, SRDX and CRISPR lines in response to different metal ions and pH changes. Figure S4: Phylogenetic analysis of HD-ZIP I subfamily members. Supplementary Table S1: Primers used in this study.

## Abbreviations

Kb	1000 base pair
MS	Murashige and Skoog medium
GF-AAS	Graphite Furnace Atomic Absorption Spectrometry
