# Supplementary material for: AtHB7/12 Regulate Root Growth in Response to Aluminum Stress
Source: Int J Mol Sci. 2020 Jun 7;21(11):4080. doi: 10.3390/ijms21114080 (PMC7312248; doi:10.3390/ijms21114080)
Supplement: Supplementary file 1 [file ijms-21-04080-s001.zip › ijms-808208 Supplementary/HB7 and HB12 Supplementary Table S1.pdf]

Supplementary Table S1. Primers used in the study.

Primer name	Primer sequence	Application
AtHB7-F	ATGACAGAAGGTGGAGAATA	Cloning to pENTR for expression vectors construction
AtHB7-R	TCATGACCAAAAATCCCGCC	
AtHB12-F	ATGGAAGAAGGAGATTTTTT	
AtHB12-R	TTATGACCAAAACTCCCACC	
AtHB7pro-F	ATATACTAGTTAACCACGACA	
AtHB7pro-R	AATGTTGTACTCAGTCTCG	
AtHB12pro-F	TTCTCATCAACTTGGCTTTC	
AtHB12pro-R	CATGGTTTTCACCAGATCTT	
AtHB7-F1	ATTGGGCTCTGCTGACATCATCGC	Crispr/Cas9 based mutations of *AtHB7*
AtHB7-R1	AAACGCGATGATGTCAGCAGAGCC	
AtHB7-F2	ATTGCGACGAGCAGATCAAGTCAC	
AtHB7-R2	AAACGTGACTTGATCTGCTCGTCG	
AtHB7-F3	ATTGAGGGTTGCAGCCGAGGCAAG	
AtHB7-R3	AAACCTTGCCTCGGCTGCAACCCT	
AtHB12-F1	ATTGTGTTTCAGCGAGATTAGTAG	Crispr/Cas9 based mutations of *AtHB12*
AtHB12-R1	AAACCTACTAATCTCGCTGAAACA	
AtHB12-F2	ATTGTAACCAAAAGAGGTTTAGCG	
AtHB12-R2	AAACCGCTAAACCTCTTTTGGTTA	
AtHB12-F3	ATTGAGGGCTGCAACCAAGACAAG	
AtHB12-R3	AAACCTTGTCTTGGTTGCAGCCCT	
AtHB7-qRT-F	GCTCAGAAAACGAAGAGAACCG	qRT-PCR
AtHB7-qRT-R	GCTCCTCAAACCCACCAAAATA	
AtHB12-qRT-F	GCAGAGACTAAACGAAGAGATG	
AtHB12-qRT-R	TCTTTCCATTATGCGACTCT	
