# Supplementary figures and images for: AtHB7/12 Regulate Root Growth in Response to Aluminum Stress
Source: Int J Mol Sci. 2020 Jun 7;21(11):4080. doi: 10.3390/ijms21114080 (PMC7312248; doi:10.3390/ijms21114080)

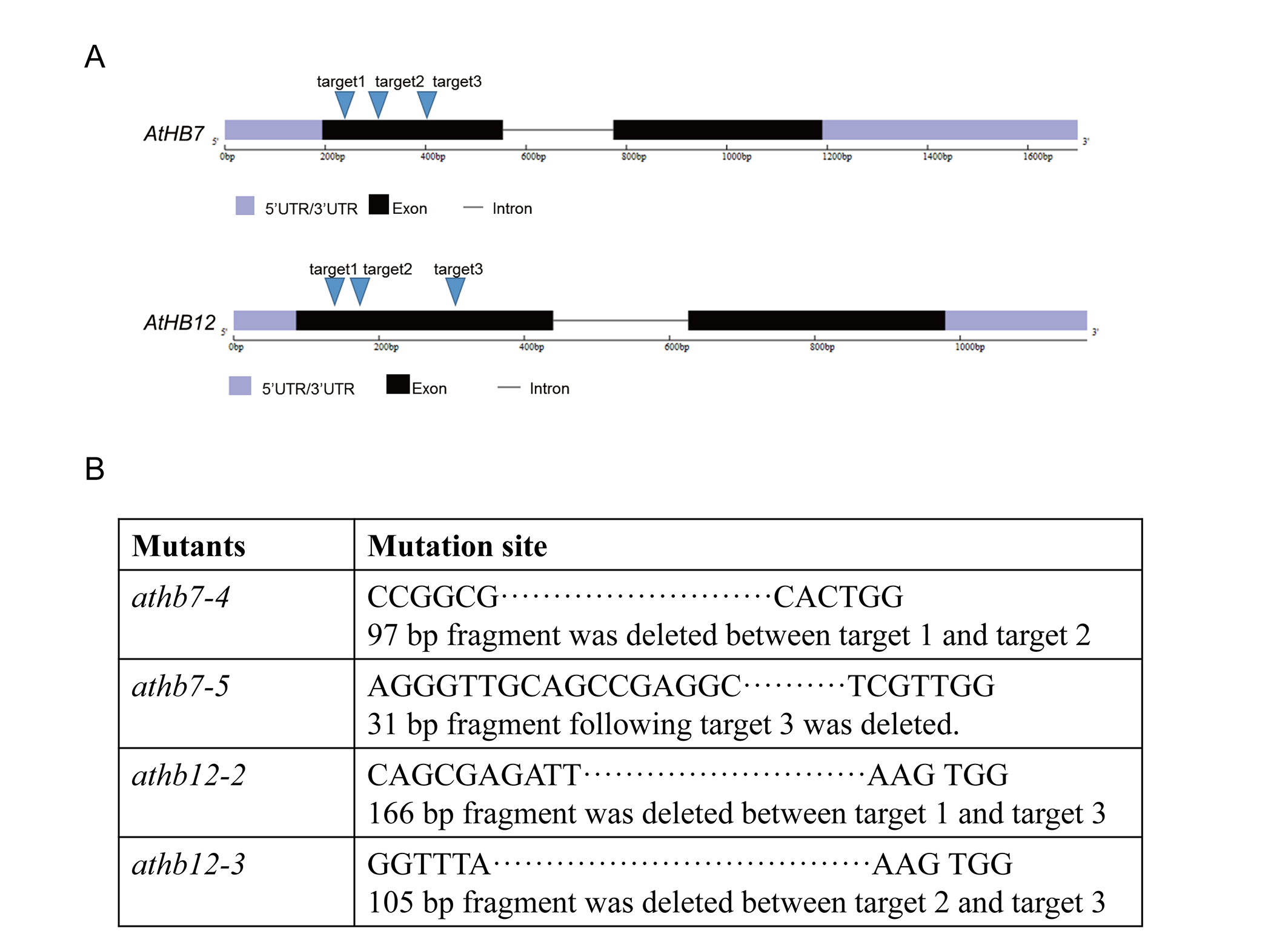

Supplement: Supplementary file 1 [file ijms-21-04080-s001.zip › ijms-808208 Supplementary/Supplementary figure S1.tif]

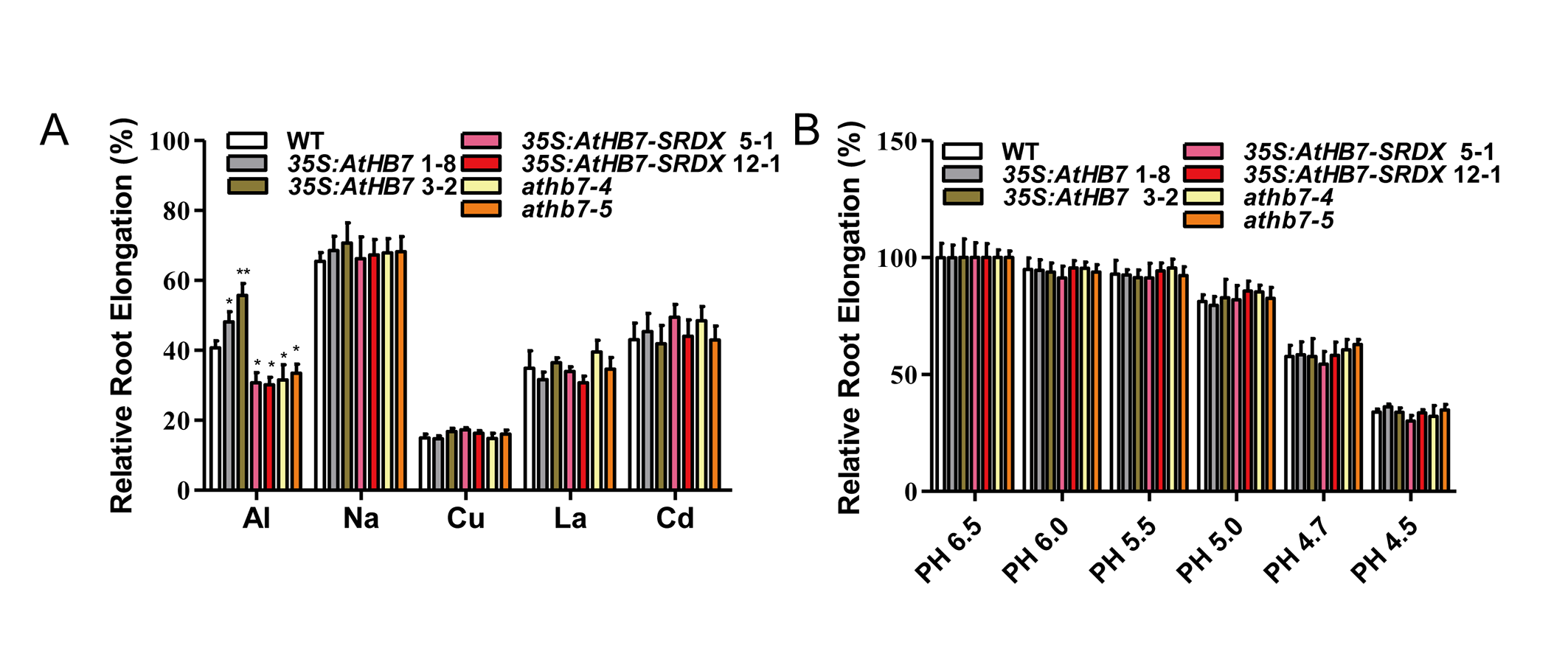

Supplement: Supplementary file 1 [file ijms-21-04080-s001.zip › ijms-808208 Supplementary/Supplementary figure S2.tif]

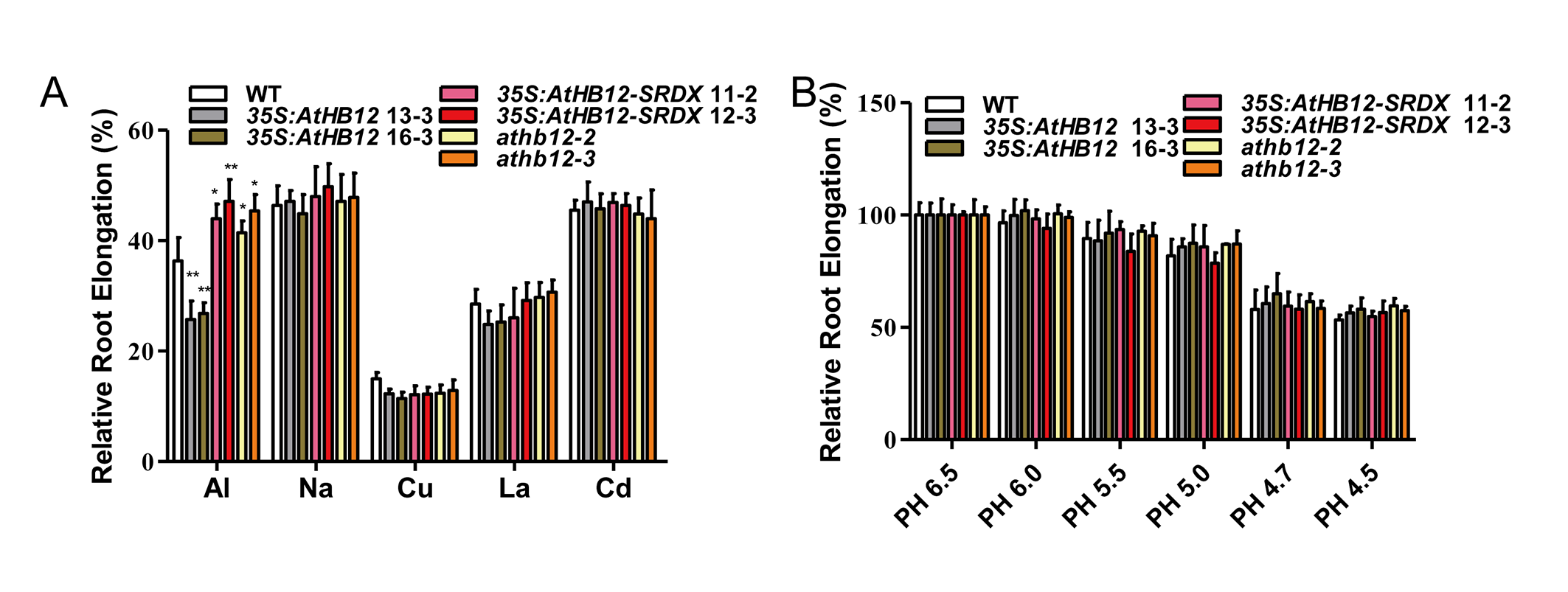

Supplement: Supplementary file 1 [file ijms-21-04080-s001.zip › ijms-808208 Supplementary/Supplementary figure S3.tif]

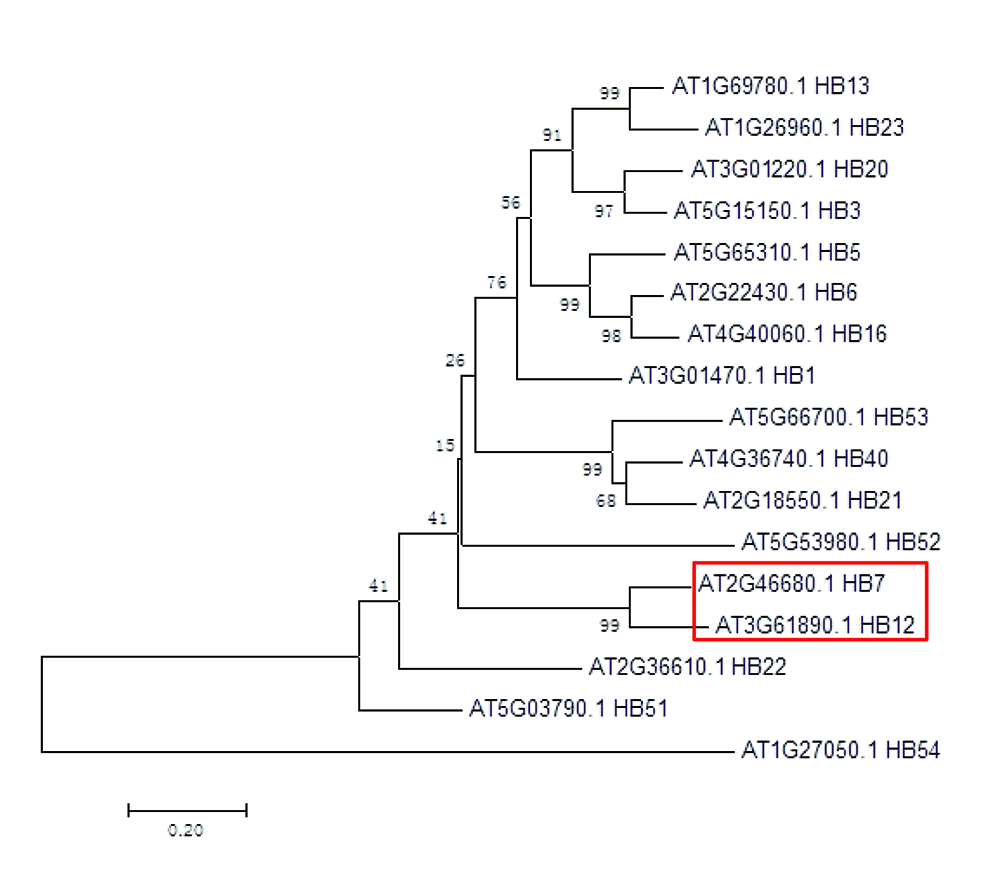

Supplement: Supplementary file 1 [file ijms-21-04080-s001.zip › ijms-808208 Supplementary/supplementary figure S4.tif]
